# Keratin 8 limits TLR-triggered inflammatory responses through inhibiting TRAF6 polyubiquitination

**DOI:** 10.1038/srep32710

**Published:** 2016-09-02

**Authors:** Xiao-Ming Dong, En-Dong Liu, Yun-Xiao Meng, Chao Liu, Ya-Lan Bi, Huan-Wen Wu, Yan-Chao Jin, Jing-Hui Yao, Liu-Jun Tang, Jian Wang, Min Li, Chao Zhang, Miao Yu, Yi-Qun Zhan, Hui Chen, Chang-Hui Ge, Xiao-Ming Yang, Chang-Yan Li

**Affiliations:** 1 Tianjin University, School of Chemical Engineering and Technology, Department of pharmaceutical engineering, Tianjin 300072, China; 2 State Key Laboratory of Proteomics, Beijing Proteome Research Center, Beijing Institute of Radiation Medicine, Beijing 100850, China; 3 AnHui Medical University, Hefei, 230032, China; 4 Department of Pathology, Peking Union Medical College Hospital, Chinese Academy of Medical Sciences and Peking Union Medical College, Tsinghua University, 1 Shuai Fu Yuan Hu Tong, Beijing 100730, China

## Abstract

Toll-like receptors (TLRs) have critical roles in innate immunity and inflammation and the detailed mechanisms by which TLR signaling is fine tuned remain unclear. Keratin 8 (CK8) belongs to the type II keratin family and is the major compontent of the intermediate filaments of simple or single-layered epithelia. Here we report that down-regulation of CK8 in mice enhanced TLR-mediated responses, rendering mice more susceptible to lipopolysaccharide (LPS)-induced endotoxin shock and *Escherichia coli*–caused septic peritonitis with reduced survival, elevated levels of inflammation cytokines and more severe tissue damage. We found that CK8 suppressed TLR-induced nuclear factor (NF)-κB activation and interacted with the adaptor tumor necrosis factor (TNF) receptor-associated factor 6 (TRAF6) to prevent its polyubiquitination. Our findings demonstrate a novel role of CK8 in negative regulation of TLR/NF-κB signaling and highlight a previously unidentified nonclassical function for CK8 in limiting inflammatory responses.

The interleukin-1 receptor (IL-1R)/Toll-like receptor (TLR) family plays a pivotal role in inflammation and host defense against invading microorganisms through the recognition of pathogen-associated molecular patterns (PAMPs) derived from various microbial pathogens, such as viruses, bacteria, protozoa and fungi^1^. These microbial components include bacterial lipopolysaccharide (LPS; TLR4 ligand), lipoproteins (TLR2 ligand), flagellin (TLR5 ligand), bacterial CpG DNA (TLR9 ligand), viral single-stranded RNA (TLR7 ligand) and viral double-stranded RNA (TLR3 ligand)^2^. All TLR signaling pathways culminate in activation of the transcription factor nuclear factor-kappaB (NF-κB), which controls the expression of an array of inflammatory cytokine genes^3^. After binding with ligands, TLRs and Toll/interleukin-1 receptor-like domain (TIR) recruits the adaptor proteins myeloid differentiation primary response gene 88 (MyD88) and IL-1R-associated kinase 1/4 (IRAK1/4).These two adaptor proteins can subsequently form a complex with tumor necrosis factor (TNF)-associated factor 6 (TRAF6) to trigger lysine 63 (K63) auto-polyubiquitination and activation of TRAF6 with the help of ubiquitin-conjugating (UBC) enzyme 13 (UBC13)/Uev1a^4^,^5^,^6^. Activation of TRAF6 in turn leads to activation of the downstream signaling pathway of the transcription factor NF-κB, as well as the production of proinflammatory cytokines, such as IL-6, TNFα, IFNγ and MCP^7^,^8^. NF-κB transcription factors are usually sequestered in the cytoplasm in an inactive form by molecules of the inhibitor of NF-κB (IκB) family. Activation of NF-κB involves the phosphorylation and proteolysis of the IκB proteins and the concomitant release and nuclear translocation of the NF-κB factors^9^. Negative regulation of TLR signaling is crucial to maintain immune homeostasis and inappropriate activation or overactivation of TLR signaling may result in inflammatory disorders such as septic shock or autoimmune diseases^10^,^11^,^12^,^13^. The identification of the negative regulators and details of the mechanisms by which TLR signaling is fine tuned remain to be fully elucidated.

Intermediate filaments (IFs) are major components of the cytoskeleton and nuclear envelope in most types of eukaryotic cells^14^ except in the axons of many Arthropoda^15^. The keratin subfamily, which is preferentially expressed in epithelia cells, has over 20 members (Keratin 1–20) that form obligate noncovalent heteropolymers of at least one type I (Keratin 9–20) and one type II keratin (Keratin 1–8)^16^. Keratin 8 and 18 (CK8/18) are the major components of the IFs of simple or single-layered epithelia found in the gastrointestinal tract, liver, exocrine pancreas, and mammary gland, from which many carcinomas arise^17^. Gene targeting techniques have been used to elucidate the function of CK8/CK18. CK8 knockout mice in one strain died around embryonic day 12 from undetermined tissue damage^18^, whereas in a different strain, homozygous CK8 FVB/N mice were shown to develop colonic hyperplasia, colitis, rectal prolapse^19^ and spontaneous chronic T helper type 2 colitis, but these phenotypes are largely absent from CK8^+/−^ mice^20^. Only 3% of heterozygous suffered from the anorectal prolapse and about 50% of homozygous CK8 null mice showed embryonic lethality. CK18 null mice were fertile, whereas old CK18 null mice (18-month-old) developed a distinct liver pathology with abnormal hepatocytes containing CK8-positive aggregates that resembled Mallory bodies seen in human livers with alcoholic hepatitis^21^.

A report from Caulin’s^22^ supports a role of CK8 in the regulation of TNFα-mediated NF-κB signaling pathway. Normal and malignant epithelia cells deficient in CK8 are ~100 times more sensitive to tumor necrosis factor (TNF)-induced cell death^22^. CK8 binds to the cytoplasmic domain of TNF receptor type 2 (TNFR2) and moderates the TNF-dependent activation of NF-κB transcription factor. However, it is as yet unknown whether CK8 plays a role in TLR/NF-κB signaling.

In this report, we show that CK8 functions as a negative regulator of TLR/NF-κB signaling by inhibiting TRAF6 polyubiquitination. CK8^+/−^ mice showed higher sensitivity to LPS-induced endotoxin shock and *E.coli*-caused septic peritonitis with increased mortality, suggesting a crucial role of CK8 in TLR-induced inflammatory response. Our study has identified a previously unrecognized role for CK8 in controlling NF-κB signaling by inhibiting polyubiquitination of TRAF6, thus negatively regulating the TLR-mediated inflammatory response.

## Results

### CK8^+/−^ mice showed higher sensitivity to LPS-induced endotoxin shock

**Figure 1:**
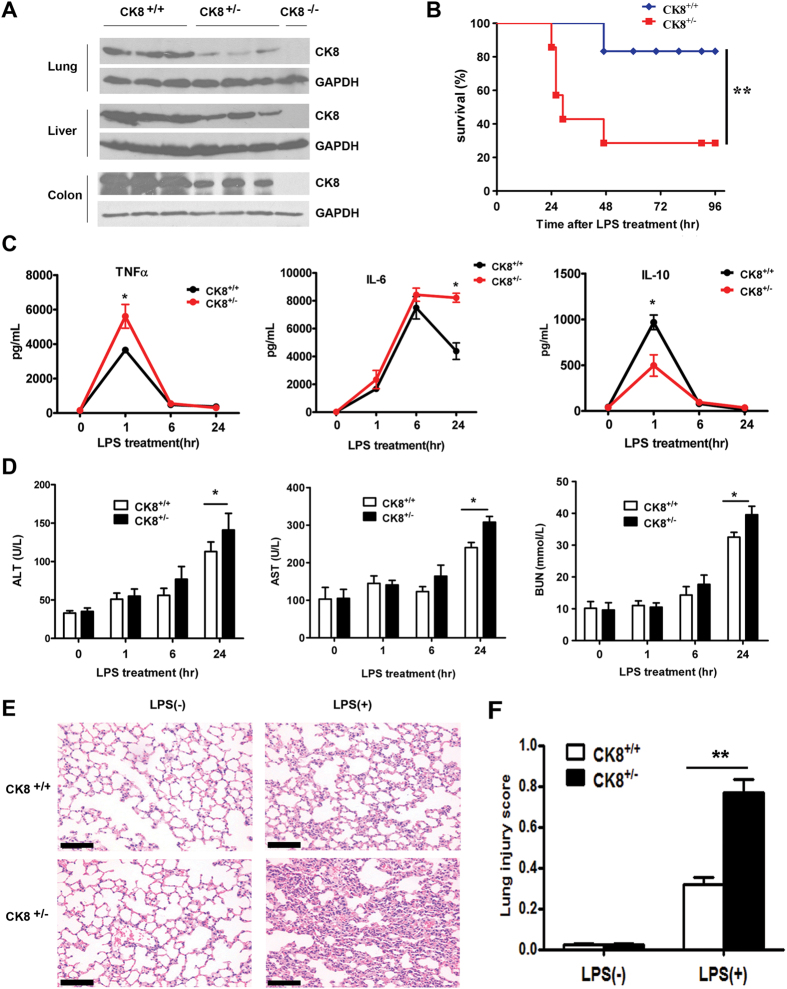
CK8^+/−^ mice showed higher sensitivity to LPS-induced endotoxin shock. (**A**) Liver, lung and colon tissue extracts from CK8^+/+^, CK8^+/−^ and CK8^−/−^ mice were prepared and the expression level of CK8 was analyzed by Western Blotting. GAPDH was used as internal control. (**B**) Survival rate of CK8^+/+^ (n = 6) and CK8^+/−^ (n = 7) mice that injected intraperitoneally with LPS at a dose of 25 mg/kg. Survival Differences were evaluated with the Mantel-Cox test. *P < 0.05, **P < 0.01, Mantel-Cox test. (**C**) Concentration of TNFα, IL-6 and IL-10 in sera from CK8^+/+^ and CK8^+/−^ mice at various times after intraperitoneal injection of LPS (20 mg/kg). (**D**) The levels of ALT, AST, BUN from the sera of CK8^+/+^ and CK8^+/−^ mice were determined after LPS injection. (**E**) HE staining of lungs from CK8^+/+^ and CK8^+/−^ mice at 24 h after LPS challenge. Original magnification: x200, scale bar: 50 μm. (**F**) The lung injury scores were assessed in 20 fields (x400 magnification) of lung sections (n = 4 per group). All data are shown as the mean ± s.d and are representative of three independent experiments. *P < 0.05, **P < 0.01.

To determine the effects of CK8 on the regulation of TLR-induced inflammation responses in CK8^+/−^ mice, we initially measured protein expression level of CK8 in homogenates of colon tissue from CK8^+/+^ (wild type), CK8^−/−^ and CK8^+/−^ mice. As expected, CK8 was completely abolished in CK8^−/−^ mice, and CK8^+/−^ mice have about 50% of the CK8 level exhibited by CK8^+/+^mice in different tissue (Fig. 1A).

CK8^+/+^ and CK8^+/−^ mice were then challenged with a lethal dose of LPS^23^ (25 mg/kg) and monitored the survival. We found that 60% of CK8^+/−^ mice died within 30 hours after LPS treatment, while 100% of CK8^+/+^ mice were alive. At 96 hours after treatment, only 30% of CK8^+/−^ mice ultimately survived and in control group, 80% of CK8^+/+^ mice survived (P < 0.01, Fig. 1B). We next analyzed the abundance of various cytokines in the serum of CK8^+/+^ and CK8^+/−^ mice after injection with LPS. We found that CK8^+/−^ mice produced more TNFα at 1 h and IL-6 at 24 h, and lower levels of IL-10 at 1 h than CK8^+/+^ mice (P < 0.05, Fig. 1C). Previous studies suggest that exposure to a high dose of LPS also causes multi-organ system failure such as lung, liver, and kidney injury^24^,^25^. We then investigated whether the liver and the kidney of the mice were injured after LPS infection. The levels of aspartate aminotransferase (AST) and alanine aminotransferase (ALT) (markers of liver injury) were higher in the sera of CK8^+/−^ mice at 24 h after LPS treatment (P < 0.05, Fig. 1D). Additionally, the blood urea nitrogen (BUN, an indicator of kidney damage) levels were elevated in the sera of CK8^+/−^ mice at 24 h after LPS treatment (P < 0.05, Fig. 1D). Thus, damage of multiple organs might cause the death of the CK8^+/−^ mice after LPS infection. The lungs of CK8^+/−^ mice were infiltrated with more inflammatory cells and exhibited a thicker alveolar septum than those of CK8^+/+^ mice after LPS challenged (Fig. 1E,F).

Thus, these data demonstrated that TLR-triggered inflammatory responses were greater in CK8^+/−^ mice, which indicated a suppressive role for CK8 in the TLR response.

### CK8^+/−^ mice showed higher sensitivity to *E.coli*-induced septic peritonitis

**Figure 2:**
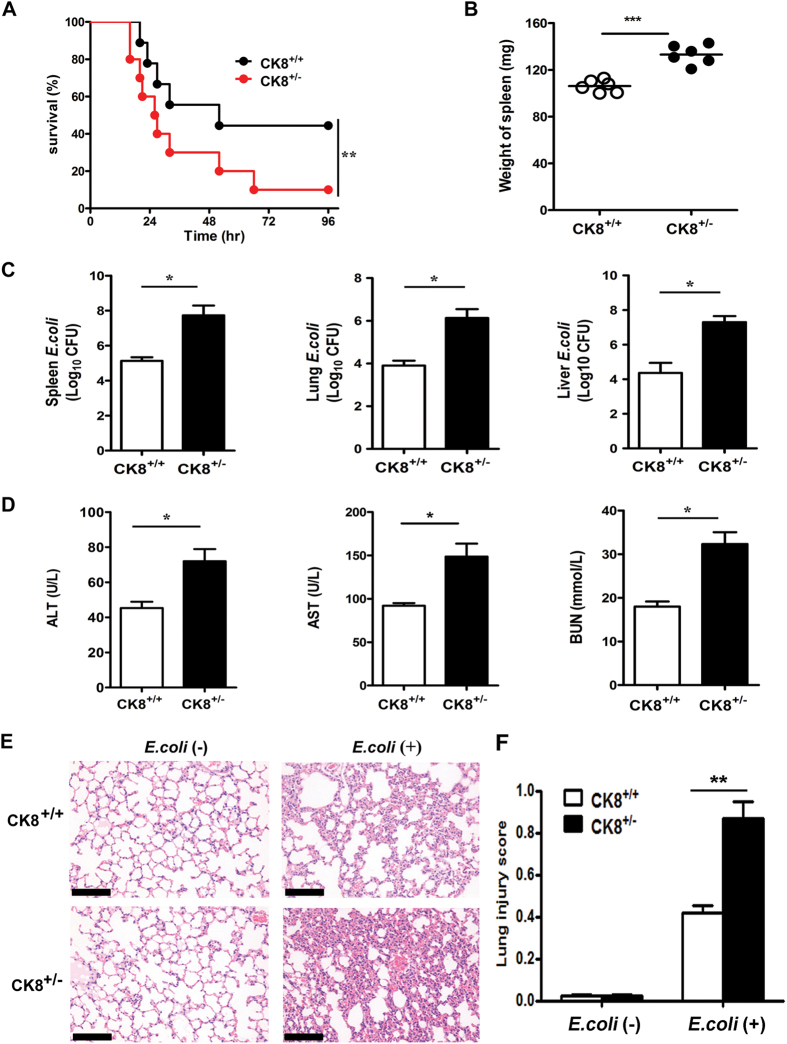
CK8^+/−^ mice showed higher sensitivity to *E.coli*-induced septic peritonitis. (**A**) Survival rate of CK8^+/+^ (n = 10) and CK8^+/−^ (n = 10) mice after intraperitoneal injection of 5 × 10^7^ *E.coli*. Survival Differences were evaluated with the Mantel-Cox test. *P < 0.05, **P < 0.01, Mantel-Cox test. (**B**) Weight of the spleens from CK8^+/+^ or CK8^+/−^ mice (n = 6 for each genotype) after infection as described in (**A**). (**C**) Bacterial load in the spleen, lung and livers of CK8^+/+^ or CK8^+/−^ mice (n = 6) at 20 h after injection with *E.coli*. (**D**) Serum concentrations of ALT, AST and BUN from CK8^+/+^ and CK8^+/−^ mice (n = 5) were measured after infection with *E.coli*. (**E**) HE staining of the lungs from CK8^+/+^ or CK8^+/−^ mice after infection as described in A. Original magnification: x200, scale bar: 50 μm. (**F**) The lung injury scores were assessed in 20 fields (x400 magnification) of lung sections (n = 5 per group). All data are shown as the mean ± s.d and are representative of three independent experiments. *P < 0.05, **P < 0.01, ***p < 0.001.

LPS is a major component of the outer membrane of Gram-negative bacteria, such as *Escherichia coli (E.coli*). Stimulation by LPS can lead to local infections or inflammatory processes through the Toll-like receptor 4 (TLR4)-mediated signalling pathway^26^,^27^. To confirm the involvement of CK8 in regulating TLR-mediated septic shock, we challenged mice with Gram-negative *Escherichia coli (E.coli*) via rectal enema. Following infection with *E.coli* for 36 hours, 90% of CK8^+/−^ mice died due to a fatal shock by bacteria, whereas 60% of the wild type (WT) mice died after *E.coli* infection (P < 0.05, Fig. 2A). The CK8^+/−^ mice developed more severe splenomegaly after bacterial infection (P < 0.001, Fig. 2B). The bacterial loading amounts of the livers, spleens and lungs of the CK8^+/−^ mice were significantly higher than those of the CK8^+/+^ mice (P < 0.05, Fig. 2C). The levels of AST, ALT and BUN were all higher compared to CK8^+/+^ mice after bacterial infection (P < 0.05, Fig. 2D). Histological analysis of the lungs showed thicker alveolar septa and dramatic pulmonary microvascular failure after *E.coli* infection (Fig. 2E,F). These results suggest that CK8^+/−^ mice are more susceptible to bacterial infection.

### CK8 is a negative regulator of TLR-induced NF-κB signaling

**Figure 3:**
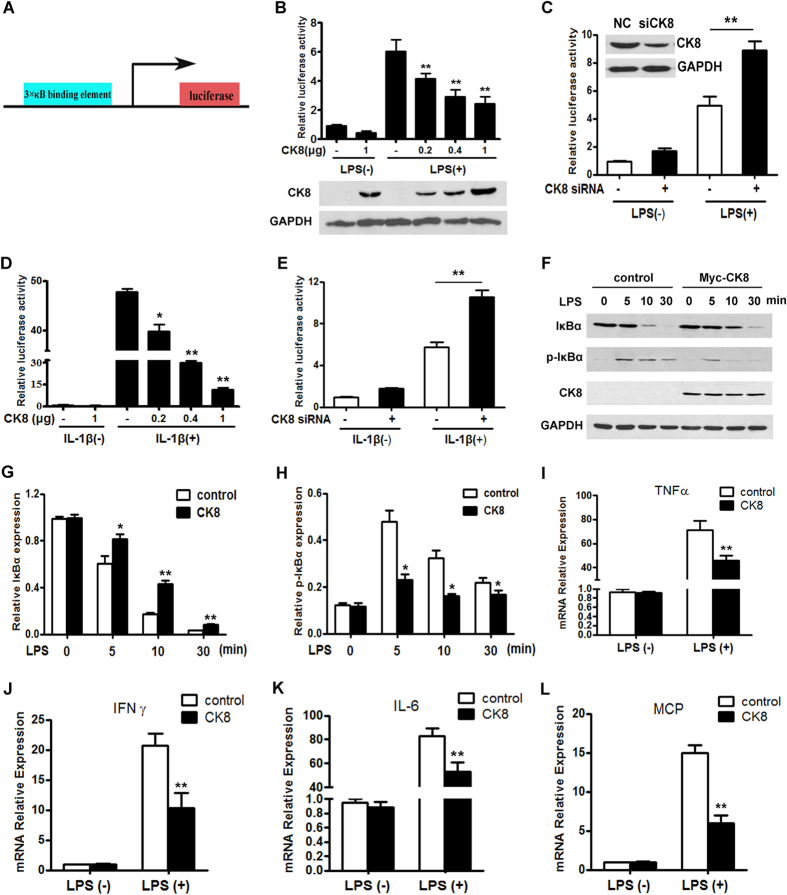
CK8 is a negative regulator of LPS or IL-1β-induced NF-κB signaling. (**A**) Schematic representation of NF-κB luciferase construct was shown. (**B**) Luciferase reporter assay of NF-κB activity after LPS (1 μg/ml) stimulation with cotransfection with increasing amounts of CK8 expression vector in HT29 cells. (**C**) HT29 cells were transfected with scramble control or CK8 siRNA and the NF-κB activity was examined after LPS treatment for 24 h using luciferase reporter assay. (**D**) NF-κB activity after IL-1β (100 ng/ml) stimulation with cotransfection with increasing amounts of CK8 expression vector in HT29 cells. (**E**) HT29 cells were transfected with scramble control or CK8 siRNA and the NF-κB activity was examined after LPS treatment for 24 h using luciferase reporter assay. (**F**) Western blotting analysis of p-IκBα, IκBα, and CK8 in lysates of HT29 cells transfected with empty vector or Myc-CK8 after treatment with LPS (1 μg/ml) for the indicated times. GAPDH was used as internal control. (**G**,**H**) Densitometry analysis of IκBα and p-IκBα level was performed by Image J software. The Western blot were performed at least three times. (**I**–**L**) HT29 cells were transfected with empty vector or CK8 expression vector and treated with LPS (1 μg/ml) for 24 h. The mRNA levels of *IL-6*, *TNFα*, *IFNγ* and *MCP* were measured using Realtime PCR. *p < 0.05, **p < 0.01.

Toll-like receptors (TLRs) have been established to play an essential role in the activation of innate immunity by recognizing specific patterns of microbial components^28^ such as LPS and cytokines such as IL-1β. All TLR signaling pathways culminate in activation of NF-κB, which controls the expression of an array of inflammatory cytokine genes. Using a NF-κB luciferase reporter vector (Fig. 3A), we found that LPS treatment induced a significant activation of NF-κB in human colon cancer cell line HT29 cells, and overexpression of CK8 significantly attenuated the NF-κB activation that was induced by LPS in a dose-dependent manner (Fig. 3B). Conversely, knockdown of endogenous CK8 using siRNA enhanced LPS-induced NF-κB activation, whereas a scrambled control siRNA did not affect its activity (Fig. 3C). The same result was obtained in the regulation of IL-1β-induced NF-κB activation (Fig. 3D,E). We further analyzed the NF-κB signaling component IκB and phosphorylation of IκB. As shown in (Fig. 3F–H), CK8 overexpression led to decreased phosphorylation and impaired degradation of IκB after LPS treatment. We also examined the effect of CK8 overexpression on the transcription of TLR/NF-κB downstream target genes *IL-6*, *TNFα*, *IFNγ* and *MCP*. These results suggest that the transcription of these genes were significantly inhibited by CK8 after LPS treatment (Fig. 3I-L).

**Figure 4:**
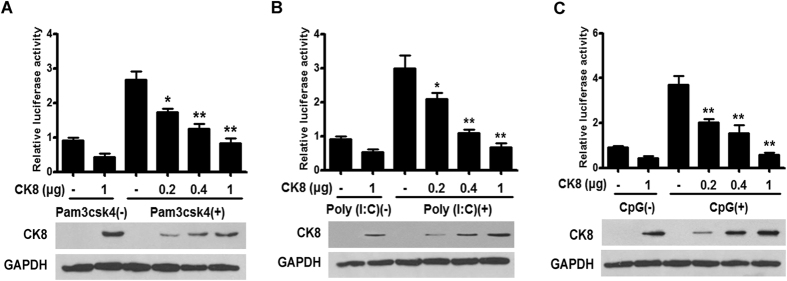
CK8 is a negative regulator of TLRs-induced NF-κB signaling. (**A**–**C**) HT29 cells were transfected with increasing amounts of CK8 expression vector and treated with the indicated TLRs inducers (Pam3csk4, 10 μg/ml; Poly (I:C), 10 μg/ml, and CpG, 10 μg/ml) for 24 h. NF-κB activity was measured using luciferase reporter assay. *p < 0.05, **p < 0.01.

To investigate the effect of CK8 overexpression on various TLRs inducers (including TLR3 inducer Poly (I:C), TLR9 inducer CpG, and TLR1/2 inducer Pam3csk4), we performed the luciferase reporter gene assay. As shown in Fig. 4A–C, overexpression of CK8 significantly attenuated the NF-κB activation induced by these inducers in a dose-dependent manner in HT29 cells. These results demonstrate that CK8 negatively regulates TLRs-induced NF-κB signaling.

### TLR-induced NF-κB signaling activation was increased in CK8^+/−^ mice

**Figure 5:**
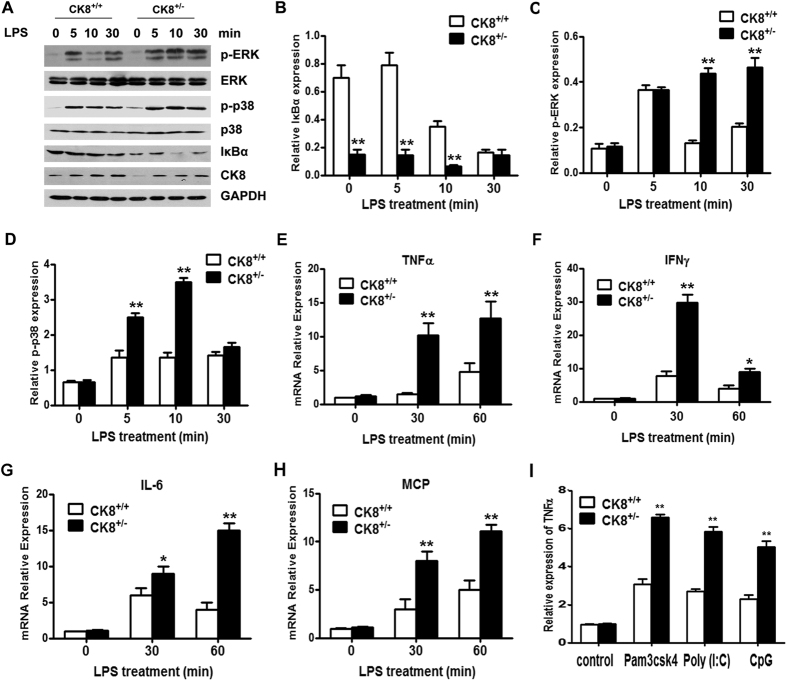
TLR-induced NF-κB signaling activation was increased in CK8^+/−^ mice. (**A**) Colon tissues from CK8^+/+^ and CK8^+/−^ mice were treated with LPS (10 μg/ml) for the indicated time and the levels of p-ERK, ERK, p-p38, p38, IκBα, and CK8 were analyzed using Western blotting analysis. GAPDH serves as internal control. (**B**–**D**) Quantitative comparison of signaling activation between CK8^+/+^ and CK8^+/−^ mice by density scanning of the blots in (**A**). The Western blot were performed at least three times. *p < 0.05, **p < 0.01. (**E**–**H**) Total RNA were extracted and the mRNA levels of *TNFα*, *IL-6*, *IFNγ* and *MCP* were measured using Realtime PCR. Results were normalized to the expression of GAPDH and are presented relative to those of untreated cells. *p < 0.05, **p < 0.01. (**I**) Colon tissues from CK8^+/+^ and CK8^+/−^ mice were treated with the indicated TLRs inducers (Pam3csk4, 20 μg/ml; Poly (I:C), 50 μg/ml, and CpG, 50 μg/ml) for 8 h, the mRNA level of *TNFα* was measured using Realtime PCR.

**Figure 6:**
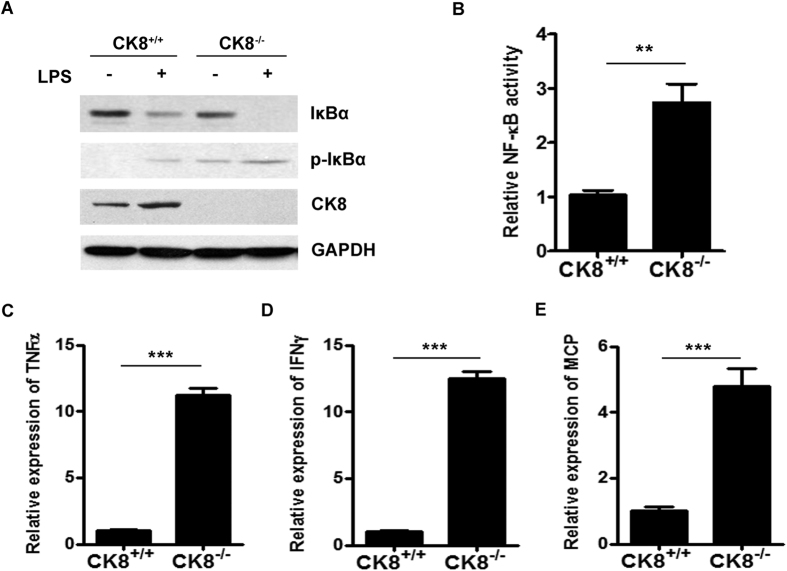
TLR-induced NF-κB signaling activation was increased in CK8^−/−^ mice. (**A**) Colon tissues from CK8^+/+^ and CK8^−/−^ mice were treated with LPS for 10min and the expression levels of p-IκBα, IκBα, and CK8 was analyzed using Western blot. GAPDH was used as internal control. (**B**) NF-κB activity was measured in colon tissues from CK8^+/+^ and CK8^−/−^ mice. (**C**–**E**) The mRNA levels of *TNFα*, *IFNγ* and *MCP* in colon tissue from CK8^+/+^ or CK8^−/−^ mice were measured using Realtime PCR. Results were normalized to the expression of GAPDH. *p < 0.05, **p < 0.01, ***p < 0.001.

LPS-triggered TLR4 signaling usually involves two different pathways: the p38 mitogen-activated protein kinase (MAPK) signaling pathway and the NF-κB signaling pathway. We further investigated the function of CK8 in regulating TLR signaling in colon explants. Colon explants from CK8^+/+^ or CK8^+/−^ mice were treated with LPS for the indicated time and the activation of MAPK and NF-κB pathways were evaluated. As shown in Fig. 5A–D, the protein level of IκBα was reduced dramatically in CK8^+/−^ colon compared to that of CK8^+/+^ mice, suggesting an abnormal activation of NF-κB in the CK8^+/−^ mice. In accordance, phosphorylation of p38 and ERK were much higher in CK8^+/−^ colon after LPS treatment. The transcription of TLR/NF-κB downstream target genes *IL-6*, *TNFα*, *IFNγ* and *MCP* were significantly increased in CK8^+/−^ mice colon compared to CK8^+/+^ mice after LPS treatment (Fig. 5E–H). Moreover, after treatment with other TLR inducers, the expression level of TNFα in CK8^+/−^ mice colon were also higher than that in CK8^+/+^ mice colon (Fig. 5I). To determine whether CK8^−/−^ mice have constitutively activated NF-κB compared to their CK8^+/+^ littermates, CK8^+/+^ and CK8^−/−^ mice were killed and the colon were isolated for western blot and NF-κB activity analysis. As shown in Fig. 6A, the protein level of IκB was apparently reduced and the phosphorylation of IκB increased in CK8^−/−^ colon tissue compared with those of CK8^+/+^ colon tissue. NF-κB activity and the downstream genes of NF-κB were much higher in CK8^−/−^ colon tissue compared to WT colon tissue (Fig. 6B–E).

These data suggest that CK8 down-regulation led to enhanced TLR-induced activation of NF-κB in mice.

### CK8 requires TRAF6 to inhibit TLR/NF-κB signaling

**Figure 7:**
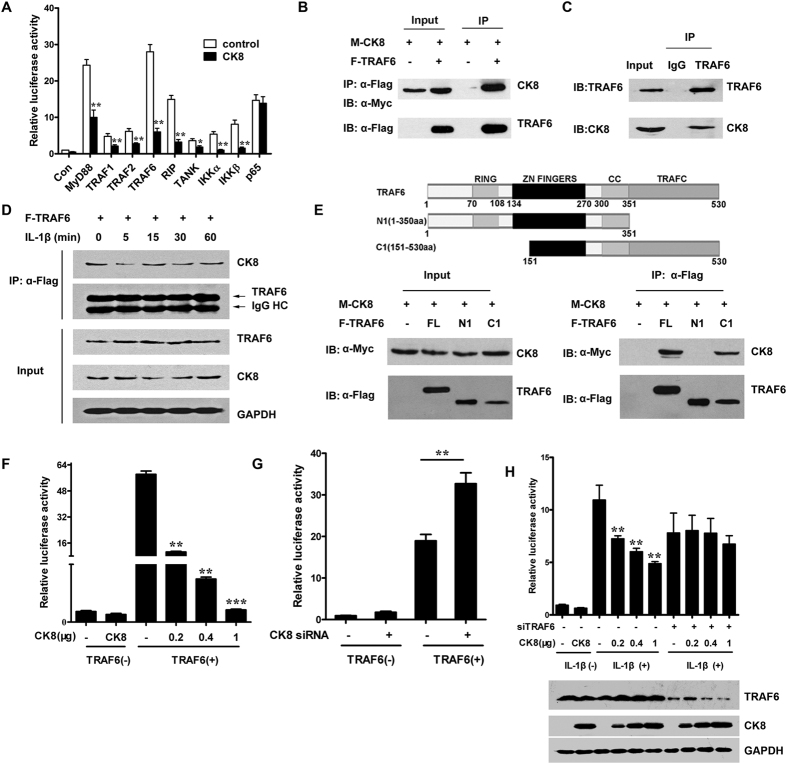
CK8 requires TRAF6 to inhibit TLR/NF-κB signaling. (**A**) NF-κB activation was measured after transfection of HEK293 cells with the indicated NF-κB activators and CK8. (**B**) Immunoblot analysis of anti-Flag immunoprecipitates of lysates from HEK293 cells that were cotransfected with Myc-CK8 and Flag-TRAF6. IB: immunoblot; IP: immunoprecipitates. (**C**) HT29 cell lysates were prepared and immunoprecipitations were performed with anti-TRAF6 antibody using normal rabbit IgG as control in the presence of RNase A and DNase I. The immunoprecipitates were analyzed with anti-CK8 antibody and anti-TRAF6 antibody. (**D**) The interaction of CK8 and TRAF6 in HT29 cells that transfected Flag-TRAF6 after IL-1β stimulation at the indicated time points. (**E**) Mapping the binding domain of TRAF6 with CK8. HEK293 cells were cotransfected with the expression plasmids as indicated. The immunoprecipitations were performed with anti-Flag antibody, and the lysates and immunoprecipitates were detected using the indicated antibodies. (**F**) HEK293 cells were co-transfected with NF-κB luciferase reporter constructs, together with TRAF6 and increasing amounts of CK8 vectors as indicated. Luciferase activity was measured 24 h after transfection. (**G**) HEK293 were cotransfected with TRAF6 and CK8 siRNA and the NF-κB activity was examined using luciferase reporter assay. (**H**) Luciferase reporter assay of NF-κB activation after IL-1β stimulation of HEK293 cells that were cotransfected with increasing amounts of CK8 and TRAF6 siRNA.*p < 0.05, **p < 0.01.

To examine the mechanism of CK8 regulation on NF-κB signaling, we investigated the effect of CK8 on NF-κB activation mediated by adaptor proteins or activators in HEK293 cells (i.e. MyD88, TRAF1, TRAF2, TRAF6, RIP1, TANK1, IKKα, and IKKβ). As shown in Fig. 7A, CK8 inhibited the NF-κB activation mediated by all these molecules, however, the NF-κB activation induced by NF-κB subunit p65 was unaffected by CK8, suggesting that CK8 negatively regulates NF-κB activation upstream of p65.

TRAF6 is a member of the TNF receptor (TNFR) associated factors (TRAFs) that mediated TNFR intracellular signaling, but unlike other TRAFs, TRAF6 also mediates LPS/TLR signaling^29^. A crucial role of TRAF6 in NF-κB signaling has been demonstrated by the fact that TRAF6 knockout cells failed to respond to IL-1β or LPS stimulation^30^. Therefore we speculated that TRAF6 is involved in the function of CK8. We first investigated whether CK8 interacts with TRAF6. Accordingly, exogenous CK8 immunoprecipitated together with exogenous TRAF6 in HEK293 cells transfected to express both CK8 and TRAF6 (Fig. 7B). Further immunoprecipitation of endogenous proteins in HT29 cells and mice colon tissue confirmed a physical association between CK8 and TRAF6 (Fig. 7C). The interaction between CK8 and TRAF6 decreased at 5min after IL-1β stimulation (Fig. 7D, Figure S1) then increased at 15min, suggesting that the interaction responded dynamically to TLR/NF-κB signaling activation.

Previous studies suggest that TRAF6 is composed of an amino-terminal RING-finger domain, a series of zinc fingers, an α-helical coiled-coil domain and a carboxy-terminal TRAF (TRAF-C) domain^31^. Subsequent domain mapping by coimmunoprecipitation revealed that the TRAF-C domain of TRAF6 mediated this interaction between CK8 and TRAF6 (Fig. 7E). Moreover, CK8 inhibited TRAF6-mediated NF-κB activation in a dose-dependent manner (Fig. 7F). Conversely, CK8 knockdown led to increased stimulatory activity of TRAF6 on NF-κB luciferase reporter (Fig. 7G). Furthermore, TRAF6 was knocked down using specific siRNA and the effect of CK8 on NF-κB signaling was investigated. The result showed that with reduced expression level of TRAF6, the inhibition effect of CK8 was attenuated significantly (Fig. 7H). Together these results indicated that TRAF6 might mediate the negative regulation of CK8 on TLR/NF-κB signaling.

### CK8 attenuates polyubiquitination of TRAF6

**Figure 8:**
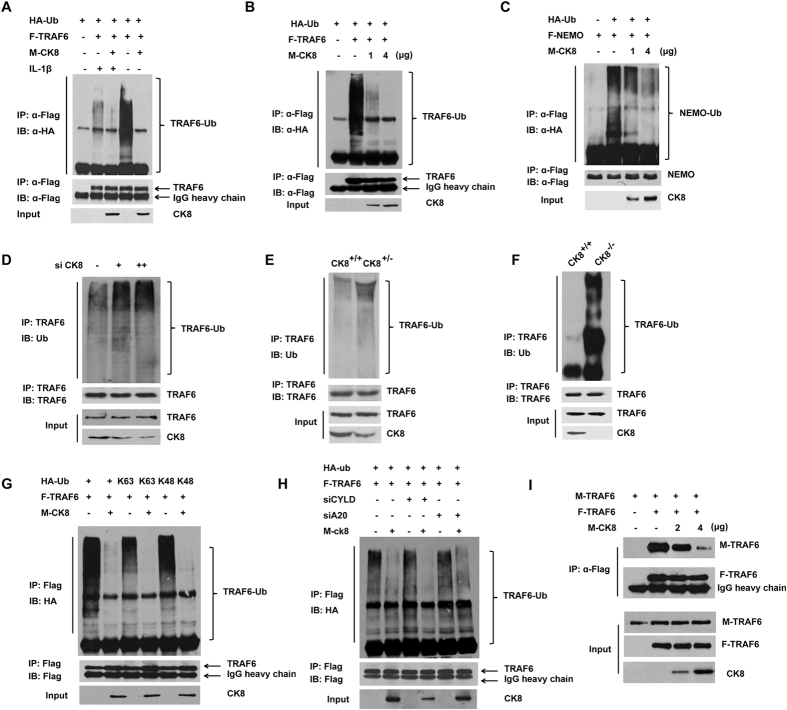
CK8 attenuates polyubiquitination of TRAF6. (**A**) HEK293 cells were co-transfected with HA-ubiquitin, Flag-TRAF6 and Myc-CK8 expression vectors as indicated. At 36 h post transfection, the proteasome inhibitor MG132 was added for 4 h. Then the indicated cells were stimulated with IL-1β for 30 min. Cells were harvested for immunoprecipitation and immunoblot analyses. Anti-HA antibody was used to detect polyubiquitinated TRAF6. (**B**,**C**) Immunoprecipitation and immunoblot analysis of HEK293 cells were transfected with the indicated plasmids and 36 h later, cells lysates were prepared for immunoprecipitation and immunoblot analysis. (**D**) HEK293 cells were transfected with various amounts CK8 siRNA followed by treatment with MG132 for 4 h and stimulation with IL-1β for 30 min. Then cells were harvested for immunoprecipitation and immunoblot analysis using the indicated antibodies. Ubiquitin antibody was used to detect endogenous polyubiquitinated TRAF6. (**E**) The colons from CK8^+/+^ or CK8^+/−^ mice at 8 weeks of age were isolated. After LPS treatment for 24 h, the ubiquitination of TRAF6 was analyzed by immunoprecipitation and Western blot with indicated antibodies. (**F**) The colons from CK8^+/+^ or CK8^−/−^ mice at 8 weeks of age were dissected. The ubiquitination of TRAF6 was analyzed by immunoprecipitation and Western blot with indicated antibodies. (**G**) HEK293 cells were transfected with the indicated plasmids, including K63 and K48 conjugation-specific ubiquitin mutants, treated with MG132 for 4 h and harvested for immunoprecipitation and immunoblot analysis. (**H**) HEK293 cells were transfected with the indicated plasmids in the presence of A20 siRNA or CYLD siRNA. Immunoprecipitation and immunoblot analysis were performed with the indicated antibodies. (**I**) HEK293 cells were transfected with Flag-TRAF6, Myc-CK8, and Myc-TRAF6 as shown, and cell lysates were immunoprecipitated with anti-Flag antibody and immunoblotted with anti-Flag or anti-Myc antibodies.

K63-polyubiquitination of TRAF6 is a key regulatory event in the activation of TRAF6^6^. We thus investigated whether CK8 affects the ubiquitination of TRAF6. As shown in Fig. 8A, polyubiquitination of Flag-TRAF6 by co-transfected HA-tagged ubiqutin was easily detected and enhanced by IL-1β stimulation. Forced expression of CK8 significantly inhibited TRAF6 polyubiqutination both in the presence and absence of IL-1β stimulation. Moreover, CK8 inhibited TRAF6 polyubiquitination in a dose-dependent manner (Fig. 8B). We further confirmed these observations by ubiquitination of NF-κB essential modulator (NEMO), a downstream substrate of TRAF6 important for the activation of IKK (Fig. 8C). Consistently, down-regulation of CK8 in HEK293 cells promoted the ubiquitination of endogenous TRAF6 with IL-1β treatment (Fig. 8D). In CK8^+/−^ mice colon, the ubiquitination of TRAF6 was also enhanced dramatically compared to that in CK8^+/+^ mice colon with LPS treatment (Fig. 8E). In CK8^−/−^ mice colon, the ubiquitination of TRAF6 was constitutively increased compared to that in CK8^+/+^ mice (Fig. 8F).

Next, we examined whether CK8 specifically affect K63-polyubiquitination of TRAF6. Transfection of conjugation-specific ubiquitin mutants showed that polyubiquitination of TRAF6 involves both K48 and K63 chains, and that CK8 inhibits both types of conjugations (Fig. 8G). Together these data indicated that CK8 inhibited the K63-polyubiquitination of TRAF6.

It has been shown that the tumor suppressor CYLD targets TRAF6 for deubiquitination to terminate TLR-triggered activation of NF-κB^32^,^33^. The zinc finger protein A20 inhibits IL-1/TLR-induced NF-κB activation by directly removing ubiquitin of TRAF6^34^,^35^. Since CK8 is not a deubiquinase, we speculate that CK8 might regulate K63-polyubiquitination of TRAF6 depending on A20 or CYLD. Then we investigated the roles A20 and CYLD in CK8-mediated inhibition of TRAF6 ubiquitination. Specific siRNAs were used to knockdown endogenous A20 or CYLD. As Fig. 8H shows, knockdown of neither A20 nor CYLD affected the inhibition effect of CK8 on TRAF6 ubiquitination, suggesting that CK8 inhibited TRAF6 ubiquitination without involving A20 and CYLD.

Homo-oligomerization of TRAF6 has been reported to be important for its subsequent polyautoubiquitination^36^,^37^. Since both amino-terminal and carboxy-terminal regions of TRAF6 contribute to its homo-oligomerization, and CK8 targeted the carboxy-terminal TRAF-C domain, we hypothesized that CK8 inhibits TRAF6 ubiquitination most probably via affecting the homo-oligomerization of TRAF6. As shown in Fig. 8I, co-immunoprecipitation analysis suggest that forced expression of CK8 inhibited the homo-oligomerization of TRAF6. Furthermore, we analyzed the TRAF6 homo-oligomerizationusing native gel assay. As Supplementary Figure S2 shows, IL-1β stimulation of cells caused a fraction of TRAF6 within cells to oliogmerize, as detected by the appearance of a TRAF6 band of reduced mobility on a native gel. Forced expression of CK8 led to a significant decrease of oligomerized TRAF6.

Taking these results together, we hypothesize that CK8 negatively regulates TLR/NF-κB signaling through inhibiting TRAF6 polyubiquitination and homo-oligomerization.

## Discussion

**Figure 9:**
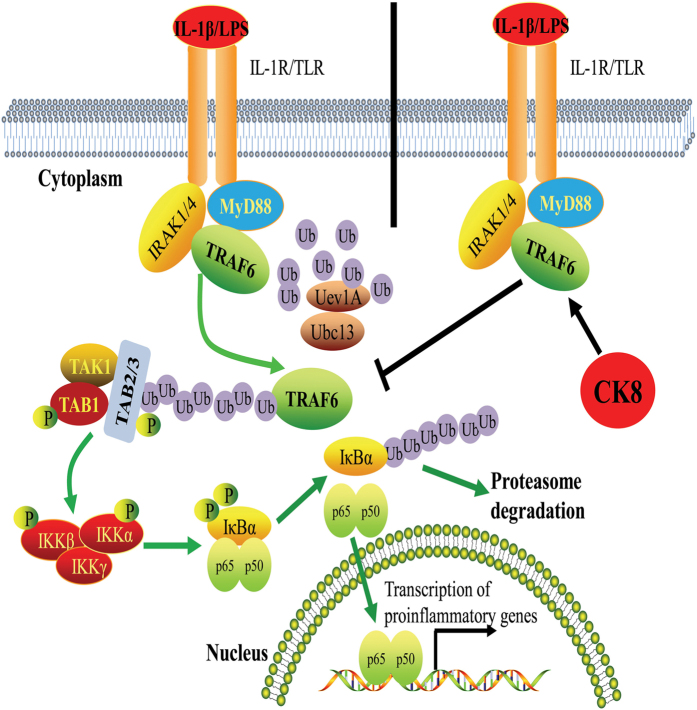
Schematic representation of negative regulation of the TLR-triggered inflammatory response pathway by CK8.

Our current study has identified CK8, a type II keratin, as a negative regulator of TLR/NF-κB signaling pathway through TRAF6. Forced expression of CK8 suppresses LPS and IL-1β-induced TLR/NF-κB signaling, and down-regulation of CK8 enhances LPS-induced NF-κB activation. CK8^+/−^ mice show higher sensitivity to LPS-induced septic shock with increased mortality, elevated levels of inflammation cytokines such as IL-6 and TNFα, and more severe tissue damage. Down-regulation of CK8 in mice leads to uncontrolled inflammatory innate responses and CK8^+/−^ mice are more susceptible to bacterial infection. Detailed mechanism analysis suggest that CK8 negatively regulates TLR-induced NF-κB signaling through interacting with TRAF6 and inhibiting TRAF6 polyubiquitination (Fig. 9). Interestingly, during activation of NF-κB signaling induced by LPS, CK8 expression level is increased (Fig. 5A), suggesting that a negative feedback loop exists in the TLR/NF-κB-CK8 axis. Our data therefore suggest that CK8 is a physiological negative regulator of TLR-induced NF-κB signaling to maintain immune homeostasis.

As an important part of the host defense system, TLR/NF-κB signaling needs to be tightly controlled to maintain immune homeostasis and avoid detrimental response. Uncontrolled signals from TLRs are involved in the pathogenesis of a variety of autoimmune diseases^38^,^39^. Deficiency in the adaptor TANK leads to overactivated TLR signaling and contributes to autoimmune pathogenesis via enhanced polyubiquitination of the ubiquitin ligase TRAF6^40^. In the present study we found that CK8 protected mice from LPS-induced septic shock and bacterial infection, which indicated that CK8 might have a role in the pathogenesis of certain infectious diseases. Previous study suggest that CK8 deficient mice develop spontaneous chronic T helper type 2 colitis (Th2 colitis)^20^ and the colonic inflammation in the CK8^−/−^ mice was prevented by administering antibiotics (vancomycin and imipenem), indicating a possible role of luminal bacteria in triggering colitis in mice with absence of CK8. However, whether negative regulation of CK8 on TLR-induced NF-κB signaling contributes to the pathogenesis of colitis needs to be further examination.

Unchecked NF-κB activity leads to unrestrained innate immune responses and a wide range of human diseases, such as septic shock, rheumatoid arthritis^41^,^42^ and tumorigenesis. So down-regulation of NF-κB signaling is as important as NF-κB activation.TRAF6 is a key signal transducer for TLR/NF-κB pathway-induced inflammation and tight regulation of TRAF6 activity is crucial for maintaining immunological homeostasis. It has been shown that K63 autopolyubiquitination of TRAF6 is essential for its activation^43^,^44^. Various negative regulators have been identified by studies targeting TRAF6 autoubiquitination such as deubiquitinases A20, CYLD, MCPIP1, USP4 or USP2a. For example, A20 is a ubiquitin-editing enzyme that serves an important role in limiting TLR signaling by targeting TRAF6^45^,^46^. In response to TLR signaling, A20 is induced and associates with TRAF6 to remove its K63-linked ubiquitin chains. CYLD negatively regulated RANK signaling by inhibiting TRAF6 ubiquitination and activation of downstream signaling events in preosteoclasts^47^. In our present study, we found that CK8 inhibited TRAF6 polyubiquitination. However, depletion of A20 or CYLD did not significantly affect the inhibitory function of CK8 on TRAF6 polyubiquitination, which indicated that CK8 regulates TRAF6 ubiquitination independently of A20 and CYLD.Homo-oligomerization of TRAF6 is essential for its subsequent autoubiquitination and several regulators have been reported to regulate TRAF6 autoubiquitination through affecting TRAF6 homo-oligomerization. TIFA induces the oligomerization and polyubiquitination of TRAF6, and then activates TAK1 and IKK^37^. MTS4, a member of the GCKIII subfamily, directly interacts with and phosphorylates TRAF6 to prevent its oligomerization and autoubiquitination^48^. Our present study suggest that CK8 interacts with TRAF-C domain of TRAF6 and inhibits TRAF6 homo-oligomerization, however, the detailed mechanism of how CK8 impairs TRAF6 homo-oligomerization remains to be investigated. Furthermore, our present study show that CK8 also inhibits K48-polyubiquitination of TRAF6. In spite of the reduction of K48-polyubiquitination, we did not detect any change in TRAF6 protein levels upon CK8 co-expression. Therefore, it remains to be established whether the change in K48-polyubiquitination has any effect on TRAF6 function.

In conclusion, our findings provide new insight into the negative regulation of TLR signaling and indicate a previously unidentified nonclassical function for CK8 in the regulation of innate inflammatory responses. As *in vivo* negative regulator of TLR/NF-κB signaling, CK8 may thus serve as a potential therapeutic target to control inflammatory diseases.

## Materials and Methods

### Mice

CK8^−/−^ FVB/N mice were purchased from Jackson Labrotary. All mice were bred in a specific pathogen-free facility, and all animal experiments were approved by and performed according to the guidelines of the Animal Ethics Committee of the Academy of Military Medical Science. The methods used were carried out in accordance with the approved guidelines.

### Western blotting Analysis

For Western blotting, cells were lysed with M-PER^®^ Mammalian Protein Extraction Reagent (Pierce, Rockford, IL, USA). Then, Western blot analysis was performed according to standard procedures. Antibodies were used at the following concentrations: CK8antibody (Abcam), 1: 1000; TRAF6antibody (Cell Signaling Technology), 1:1000; NEMO antibody (Santa Cruz), 1:1000; A20 antibody (Santa Cruz), 1:1000; CYLD antibody (Santa Cruz), 1:1000; Ub antibody (Santa Cruz), 1:500, Myc antibody (sc-40, Santa Cruz), 1:1000; Flag (F3165, Sigma), 1:5000; GAPDH (sc-47778, Santa Cruz), 1:1000; antibodies to ERK(1:500), p-ERK(1:500), p38(1:500), p-p38(1:500), IκBα(1:500) were purchased from ABclonal Technology (USA). Chemiluminescent detection was conducted using supersignal substrate (Pierce) according to the manufacturer’s specifications.

### LPS-Induced Endotoxin Shock

CK8^+/+^ and CK8^+/−^ mice were age- and sex-matched. Mice were injected intraperitoneally (i.p.) with LPS (25mg/kg body weight) at 8–10 weeks of age. Survival of the mice was monitored for up to 96 h after injection. For serum cytokine assay, sera were collected at various time points after the LPS injection. Lungs were also obtained at 24 hours for histological analysis.

### Cytokine analysis

For cytokine analysis, Orbital blood was isolated from the mice for 400 μL, followed by centrifugation at 3500 rmp × 15 min, the supernatant were detected (Mouse Inflammation Kit, BD, US) in a BD FACSCalibur. Protein extracts from mouse tissues were determined by ELISA (Boster bio- Engineering Co., Wuhan).

### Histopathological analysis

The lung samples were fixed in10% neutral buffered formalin for 1 day, subsequently routinely processed, embedded inparaffin, and stained with hematoxylin and eosin (H&E). The extent of LPS-induced injury was determined according to lung injury score as previously described^49^. The score was measured on 20 high-power fields (x400 magnification) of lung sections for each group.

### Bacterial infection

For sepsis models with bacterial species, CK8^+/+^ and CK8^+/−^ mice were injected i.p. with 5 × 10^7^ colony-forming units (CFU) of live Gram-negative *E.coli* at 8–10 weeks of age. Survival of the mice was monitored. Serum and organs (spleen, Lung, Liver) were collected and analyzed.

### Cell lines and Reagents

HEK293 cells and HT29 cells were maintained in DMEM (Gibco Invitrogen, CA) with 10% fetal calf serum (FCS). All the cells were cultured in a 37 °C incubator with 5% CO_2_ in the presence of 2 mML-glutamine, 100 IU/ml penicillin, 100 mg/ml streptomycin. LPS was purchased from Sigma (USA).Poly (I:C), CpG, and Pam3csk4 were purchased from InvivoGen (USA).

### Lentiviral vector construction and production

For construction of lentivirus-mediated RNA interference, two siRNA oligos against CK8 were synthesized in GenePharma Biotechnology, the sequences are as follows: si CK8-1: 5′-CCGTGGTTGTGAAGAAGATTT-3′ and siCK8-2: 5′-GCATGTACCAGATCAAGUATT-3′. The siRNA sequences were cloned into a psicoR-GFP vector togenerate siCK8 lentivirus. The siCK8 lentivirus expresses CMV promoter-driven GFP protein and U6 promoter-driven siRNA targetingCK8. A negative control siRNA was cloned into psicoR-GFP as a control. For production of lentivirus, HEK293 cells were cotransfected with transfer vectors pBPLV-CK8 or psicoR-GFP-siCK8 with the packaging vectors including pLP1, pLP2 and pLP-VSVG. Lentivirus were harvested 72 h after transfection, passed through a 0.45-μm filter, and concentrated 100-fold by ultracentrifugation through 20% sucrose cushion(100,000 g for 90 min; 4 °C). Titers of viral stock were determined on HT1080 cells transduced by spinoculation (1000 g for 90 min; 30 °C) and analyzed by FACS 48 h later. GFP-positive cells were sorted using fluorescence-activated cell sorter (FACS; BD Biosciences).

### Luciferase activity assay

Luciferase assays were carried out at 24 hours posttransfection with the Dual-luciferase Reporter Assay System (Promega). Transfection efficiencies were normalized using cotransfected plasmid pRL-TK measured by *Renilla* luciferase activity (Promega). Luciferase activity was measured 24 hours later.

### Quantitative real-time RT-PCR

Total RNA was reverse-transcribed and amplified using reverse transcription and PCR kits, respectively (Promega Corp., Madison, WI, US). Real-time RT-PCR was performed by Bio-Rad IQ5 (Bio-Rad, US). The abundance of mRNA of each gene was normalized to GAPDH. The sequences of the primers are provided in Supplementary Table S1.

### Organ Cultures

Colon tissue from CK8^+/+^ and CK8^+/−^ mice were cultured in serum-free DMEM containing penicillin and streptomycin. The colon tissue were harvested at the different time points after treatment with LPS. Proteins from colon tissue were extracted for western blot analysis and total RNA were extracted for RT-PCR analysis.

### Co-immunoprecipitation

Cells were washed once in PBS, and lysed in 1 ml of lysis buffer (50 mM TrisHCl, pH 7.4, 150 mM NaCl, 1 mM EDTA, 1% Triton X-100), and centrifuged for 15 min at 12,000 rpm at 4 °C. The supernatant was transferred to a fresh tube, and immunoprecipitations were performed with anti-Flag affinity gel (A2220, Sigma) or anti-Myc antibody (Santa Cruz) followed by adsorption to protein A/G plus-agarose beads (sc-2003, Santa Cruz). After SDS-PAGE, the samples were transferred onto polyvinylidenedifluoride membranes (Amersham life science) and probed with a variety of antibodies. For detecting interaction of endogenous CK8 with TRAF6, HEK293 cells were lysed in 0.5 ml lysis buffer and immunoprecipitated with anti-TRAF6 antibody or control serum (Santa Cruz). After extensive washing with the lysis buffer, the immunoprecipitates were resolved by SDS–PAGE, followed by Western blot analysis using the anti-CK8 antibody.

### Deubiquitination of TRAF6

HEK293 cells were transfected with the indicated constructs. 36 h after transfection, the cells were treated with the proteasome inhibitor MG132 (20 mM) for 4 h before being harvested and lysed in lysis buffer. Anti-HA antibody was used to detect polyubiquitinated TRAF6. For detecting the endogenous ubiquitinated of TRAF6, the colons from CK8^+/+^ or CK8^+/−^ mice at 8 weeks of age were isolated. After LPS treatment, the ubiquitination of TRAF6 was analyzed by immunoprecipitated with TRAF6 (H-274) antibody and immunoblotted with anti-Ub antibody or anti-TRAF6 antibody.

### Statistical analysis

All experiments were performed at least three times. Statistical analysis was achieved using Graphpad Prism Software. Data were reported as means ± SD and the statistical significance was assessed by one-way analysis of variance followed by Students–Newman–Keuls tests for multiple group comparison. Kaplan-Meier curves were constructed to compare survival. Differences in survival were evaluated with the Mantel-Cox test. A value of p ≤ 0.05 was considered to be significant.

## Additional Information

**How to cite this article**: Dong, X.-M. *et al.* Keratin 8 limits TLR-triggered inflammatory responses through inhibiting TRAF6 polyubiquitination. *Sci. Rep.* **6**, 32710; doi: 10.1038/srep32710 (2016).

## Supplementary Material

Supplementary Information
