# Supplementary material for: Keratin 8 limits TLR-triggered inflammatory responses through inhibiting TRAF6 polyubiquitination
Source: Sci Rep. 2016 Sep 2;6:32710. doi: 10.1038/srep32710 (PMC5009362; doi:10.1038/srep32710)
Supplement: Supplementary Information [file srep32710-s1.doc]

**Keratin 8 limits TLR-triggered inflammatory responses through inhibiting TRAF6 polyubiquitination**

**Names of authors:** Xiao-Ming Dong^1,2#^,En-Dong Liu^3#^, Yun-Xiao Meng^4,#^, Chao Liu^3^, Ya-Lan Bi, ^4^ Huan-Wen Wu^4^, Yan-Chao Jin^1^, Jing-Hui Yao^3^, Liu-Jun Tang^2^, Jian Wang^2^, Min Li^3^, Chao Zhang^2^, Miao Yu^2^, Yi-Qun Zhan^2^, Hui Chen^2^, Chang-Hui Ge^2^, Xiao-Ming Yang^1,2*^, Chang-Yan Li^2,3*^

**Authors' affiliations:**

^1^ Tianjin University, School of Chemical Engineering and Technology, Department of pharmaceutical engineering, Tianjin 300072, China

^2^ State Key Laboratory of Proteomics, Beijing Proteome Research Center, Beijing Institute of Radiation Medicine, Beijing 100850, China

^3^ An Hui Medical University, Hefei, 230032, China

^4^ Department of Pathology, Peking Union Medical College Hospital, Chinese Academy of Medical Sciences and Peking Union Medical College, Tsinghua University, 1 Shuai Fu Yuan Hu Tong, Beijing 100730, China

**Supplementary Materials and Methods**

**Native Gel Electrophoresis**

TRAF6 self-association was examined by native PAGE. HEK293T cells were seeded into 15-cm dishes (3 × 10^6^ cells) 24 h before transfection. Cells were stimulated with IL-1β 48 h after transfection and harvested by scraping. Cells were lysed for 1 h on ice in 200 μl of lysis buffer (50 mm Tris, pH 7.4, 150 mm NaCl, 30 mm NaF, 5 mm EDTA, 10% glycerol, 1 mm Na_3_VO_4_, 40 mm β-glycerophosphate, 1% Triton X-100 with 0.1 mm PMSF, 5 μg/ml leupeptin, pepstatin, and aprotinin freshly added). Whole cell lysates were clarified by centrifugation. Before sample loading, 7% polyacrylamide gels were pre-run for 60 min at 40 mA with 25 mm Tris, 192 mm glycine (pH 8.4) with and without 1% deoxycholate in the cathode and anode chamber, respectively. Cleared lysates in native sample buffer (10 mg protein, 62.5 mm Tris-Cl, pH 6.8, 15% glycerol, and 1% deoxycholate) were applied to the gels, and proteins were separated by electrophoresis for 2 h at 40 mA. Immunoblotting was performed using standard conditions.

**Supplementary Figure**

**
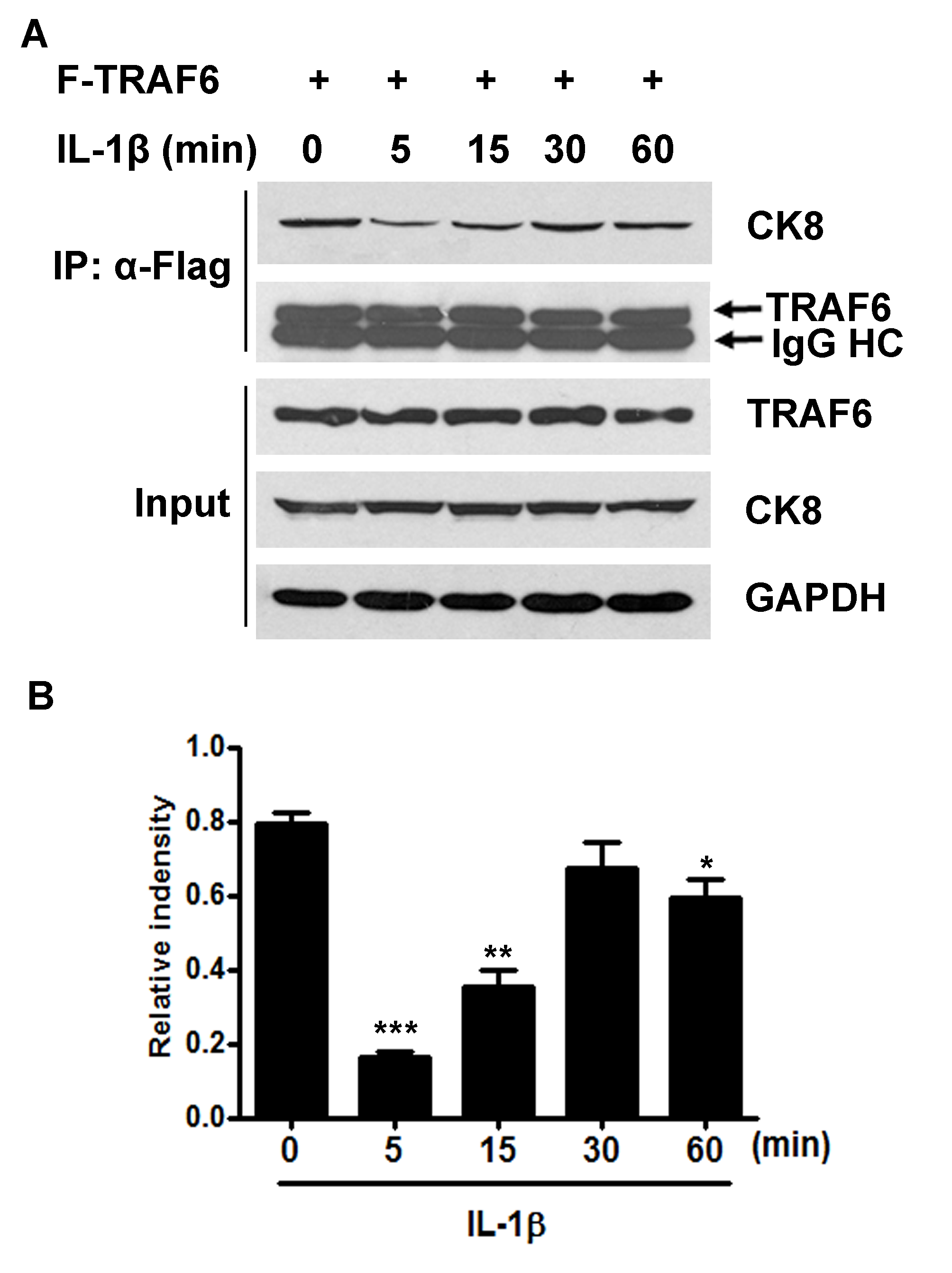
**

**Figure S1** (A) The interaction between CK8 and TRAF6 in HT29 cells that transfected Flag-TRAF6 after IL-1β stimulation at indicated time points. (B) Quantitative comparison of immunoprecipitated CK8 level by density scanning of the blots in (A), *P<0.05, **P<0.01, ***p<0.001.


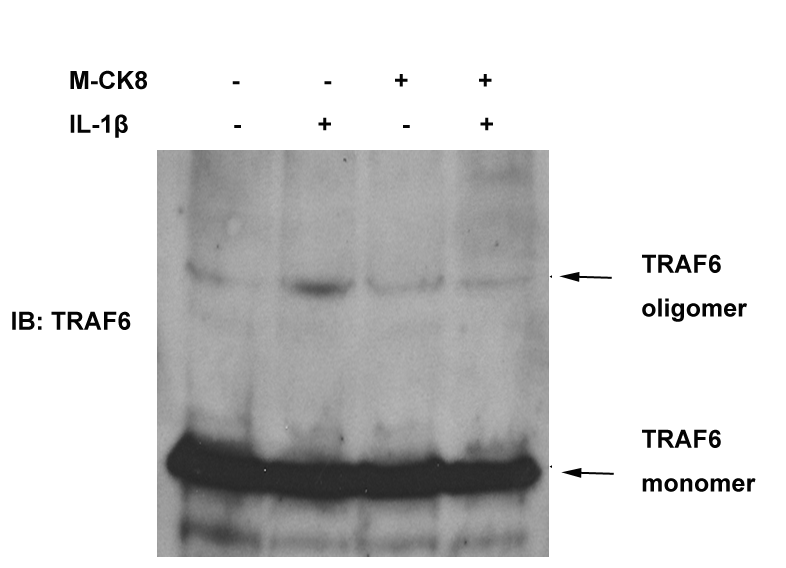


**Figure S2 CK8 inhibits TRAF6 homo-oligomerization.** HEK293 cells were transfected with CK8 overexpressing vector for 24h and then stimulated with IL-1β (100ng/ml) for 30min. Then homo-oliogomerization of TRAF6 was investigated using Native Gel Electrophoresis as the Supplementary Materials and Methods described.

**Supplementary Table S1 Real-time quantitative PCR primers**

| Primer name | Primer sepuences（5’-3’） |
| --- | --- |
| hTNFα-F  hTNFα-R  hIL6-F  hIL6-R  hIFNγ-F  hIFNγ-R  hMCP1-F  hMCP1-R  hGAPDH-F  hGAPDH-R  mTNFα-F  mTNFα-R  mIL6-F  mIL6-R  mIFNγ-F  mIFNγ-R  mMCP1-F  mMCP1-R  mGAPDH-F  mGAPDH-R | CCTCTCTCTAATCAGCCCTCTG  GAGGACCTGGGAGTAGATGAG  ACTCACCTCTTCAGAACGAATTG  CCATCTTTGGAAGGTTCAGGTTG  TCGGTAACTGACTTGAATGTCCA  TCGCTTCCCTGTTTTAGCTGC  CAGCCAGATGCAATCAATGCC  TGGAATCCTGAACCCACTTCT  TGTTGCCATCAATGACCCCTT  CTCCACGACGTACTCAGCG  GACGTGGAACTGGCAGAAGAG  TTGGTGGTTTGTGAGTGTGAG  CCAGAGATACAAAGAAATGATGG  ACTCCAGAAGACCAGAGGAAAT  GCCACGGCACAGTCATTGA  TGCTGATGGCCTGATTGTCTT  TTAAAAACCTGGATCGGAACCAA  GCATTAGCTTCAGATTTACGGGT  AGGTCGGTGTGAACGGATTTG  TGTAGACCATGTAGTTGAGGTCA |
|  |  |
